# Selectively disrupted sensorimotor circuits in chronic stroke with hand dysfunction

**DOI:** 10.1111/cns.13799

**Published:** 2022-01-10

**Authors:** FeiWen Liu, ChangCheng Chen, WenJun Hong, ZhongFei Bai, SiZhong Wang, HanNa Lu, QiXiang Lin, ZhiYong Zhao, ChaoZheng Tang

**Affiliations:** ^1^ Department of Rehabilitation Medicine Chengdu Second People's Hospital Chengdu China; ^2^ Department of Rehabilitation Medicine Qingtian People's Hospital Lishui China; ^3^ Department of Rehabilitation Medicine Nanjing Drum Tower Hospital The Affiliated Hospital of Nanjing University Medical School Nanjing China; ^4^ Yangzhi Rehabilitation Hospital Affiliated to Tongji University (Shanghai Sunshine Rehabilitation Center) Shanghai China; ^5^ Centre for Health, Activity and Rehabilitation Research (CHARR) School of Physiotherapy The University of Otago Dunedin New Zealand; ^6^ Neuromodulation Laboratory Department of Psychiatry School of Medicine The Chinese University of Hong Kong HKSAR China; ^7^ Guangzhou Brain Hospital The Affiliated Brain Hospital of Guangzhou Medical University Guangzhou China; ^8^ Department of Neurology School of Medicine Emory University Atlanta Georgia USA; ^9^ Key Laboratory for Biomedical Engineering of Ministry of Education Department of Biomedical Engineering College of Biomedical Engineering & Instrument Science Zhejiang University Hangzhou China; ^10^ Capacity Building and Continuing Education Center National Health Commission of the People's Republic of China Beijing China

**Keywords:** effective connectivity, functional reorganization, Granger causality analysis, resting‐state functional magnetic resonance imaging, stroke

## Abstract

**Aim:**

To investigate the directional and selective disconnection of the sensorimotor cortex (SMC) subregions in chronic stroke patients with hand dysfunction.

**Methods:**

We mapped the resting‐state fMRI effective connectivity (EC) patterns for seven SMC subregions in each hemisphere of 65 chronic stroke patients and 40 healthy participants and correlated these patterns with paretic hand performance.

**Results:**

Compared with controls, patients demonstrated disrupted EC in the ipsilesional primary motor cortex_4p, ipsilesional primary somatosensory cortex_2 (PSC_2), and contralesional PSC_3a. Moreover, we found that EC values of the contralesional PSC_1 to contralesional precuneus, the ipsilesional inferior temporal gyrus to ipsilesional PSC_1, and the ipsilesional PSC_1 to contralesional postcentral gyrus were correlated with paretic hand performance across all patients. We further divided patients into partially (PPH) and completely (CPH) paretic hand subgroups. Compared with CPH patients, PPH patients demonstrated decreased EC in the ipsilesional premotor_6 and ipsilesional PSC_1. Interestingly, we found that paretic hand performance was positively correlated with seven sensorimotor circuits in PPH patients, while it was negatively correlated with five sensorimotor circuits in CPH patients.

**Conclusion:**

SMC neurocircuitry was selectively disrupted after chronic stroke and associated with diverse hand outcomes, which deepens the understanding of SMC reorganization.

## Graphical abstract

Stroke remains the primary reason for adult disability, and hand function recovery is vital for survivors to regain quality of life. Here, we found two functional reorganization patterns in chronic stroke patients with different hand outcomes. For stroke patients with a partially paretic hand (PPH), all motor‐related sensorimotor circuits were positive, while for stroke patients with a completely paretic hand (CPH), this relationship was reversed. Meanwhile, the effective couplings among the PSC_1, inferior temporal gyrus, precuneus, and postcentral gyrus were correlated with hand performance in all stroke patients. PMC, primary motor cortex; PSC, primary somatosensory cortex.

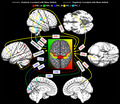

## 1 INTRODUCTION

Stroke remains the primary reason for adult disability,^1^ and hand function recovery is vital for survivors to regain functional independence.^2^ Motor outcomes following stroke have been found to be associated with infarction size,^3^ lesion topography,^4^, ^5^ gray matter plasticity,^6^ corticospinal tract integrity,^7^ functional network connectivity,^8^, ^9^ and frequency‐specific local oscillations.^10^ Benefiting from these neuroimaging discoveries in stroke populations, recent studies have suggested that modulating key sensorimotor nodes by noninvasive brain stimulation^11^, ^12^, ^13^, ^14^, ^15^ could promote motor recovery after stroke. Hence, neuroimaging opens the door for understanding the pathophysiology of motor deficits following stroke and may inspire progress in personalized, neurobiologically informed neuromodulation.^16^, ^17^

Resting‐state functional magnetic resonance imaging (fMRI) can non‐invasively explore intrinsic human brain activity,^18^ and connectivity analysis provides an effective framework for understanding information interactions among brain regions after stroke.^19^ In well‐recovered stroke patients, although activation patterns are close to those in healthy controls, the network connectivity is aberrant.^20^ Plastic changes in functional connectivity throughout the sensorimotor regions have been demonstrated to be associated with upper extremity dysfunction and recovery following stroke.^16^, ^21^ In cross‐sectional studies, disrupted interhemispheric sensorimotor connectivity was positively correlated with motor dysfunction,^22^ while it was inversely affected by the lesion load of the corticospinal tract.^23^, ^24^ In longitudinal studies, connectivity between the ipsilesional primary motor cortex (PMC) and contralesional supplementary motor area (SMA) in the early days could predict long‐term motor outcomes after stroke,^25^ and dynamic connectivity changes in the cerebrocerebellar circuits were accompanied by spontaneous recovery in stroke patients with pontine^26^ or subcortical^27^ infarcts. In neurorehabilitation studies, regulating the bilateral PMC through bihemispheric transcranial direct current stimulation,^15^ priming the ipsilesional PMC through intermittent theta burst stimulation,^11^ and inhibiting the contralesional PMC through repetitive transcranial magnetic stimulation^13^ could facilitate motor recovery following stroke. However, the direction of functional interactions between the sensorimotor cortex (SMC) and whole brain following stroke is less clear.

Effective connectivity (EC) is mostly employed for task‐evoked fMRI data, including dynamic causal modeling, psychophysiological interactions, structural equation modeling, and Granger causality analysis.^28^ In contrast to the non‐directional characteristic of functional connectivity, EC analysis can delineate the causal influences among brain regions. Using dynamic causal modeling,^13^, ^29^, ^30^, ^31^ several milestone studies have investigated EC among key sensorimotor areas following stroke. Rehme et al. reported that the interhemispheric coupling between the bilateral PMC was associated with illness duration^29^ and the severity of deficits.^30^ Grefkes et al. found that inhibiting the contralesional PMC by transcranial magnetic^13^ or noradrenergic^31^ stimulation increased the improvement‐associated couplings from the ipsilesional SMA to the PMC. Using structural equation modeling, Sharma et al. found that influence from the contralesional prefrontal cortex to the SMA was correlated with better motor performance during motor imagery in well‐recovered survivors.^20^ Another study investigating resting‐state EC among the frontoparietal area and sensorimotor system found that stroke patients showed decreased influences from the superior parietal lobule to both the PMC and SMA in the lesioned hemisphere.^32^ However, as a data‐driven exploratory method, Granger causality analysis has rarely been used in stroke studies. The SMC involves a wide spectrum of integrated motor functions and can be divided into seven subregions in each hemisphere.^33^ Given the different functions of SMC subregions and their associations with motor deficits after stroke, we investigated whether the resting‐state EC patterns of the SMC subregions suffer selective disruption in chronic subcortical stroke patients with hand dysfunction.

Here, we first defined the seven SMC subregions on the basis of the probabilistic cytoarchitectonic atlases for each hemisphere and then calculated the whole‐brain resting‐state fMRI EC patterns for each SMC subregion in each participant. Next, we examined EC differences between all stroke patients and healthy participants and between stroke subgroups with different hand outcomes. Finally, brain‐behavior correlations between EC patterns and hand performance were also explored.

## 2 METHODS

### 2.1 Recruitment of participants

Our project was approved by the Hospital Ethics Committee (2014 Interim Review No. 279) and was conducted in line with the Helsinki requirements. Before enrollment, each subject was notified and signed an informed consent form. In this study, we collected 65 chronic stroke patients with left subcortical infarction/hemorrhage and 40 healthy controls. Patients satisfying the following criteria were recruited: (a) first‐episode stroke with a lesion mainly involved in the left subcortical nuclei (eg, basal ganglia and thalamus); (b) aged 30–80 years; (c) course of disease ≥3 months; (d) motor impairments of the upper limb and hand as evaluated by the Fugl‐Meyer scale; and (e) dextromanuality as evaluated by the Edinburgh Handedness Inventory. We excluded those patients from this study who had MRI contraindications, severe cognitive impairment/aphasia/neglect, and unstable illness states (eg, serious atrial fibrillation and multiple organ failure). Healthy participants who had no neuropsychiatric history or cognitive impairments were recruited from the local community.

As per previous studies,^8^, ^10^ we used the Paretic Hand Scale (see Supporting Materials) to divide stroke patients into the partially (PPH) and completely (CPH) paretic hand subgroups. This scale was specifically designed to evaluate the practical function of the hand in everyday life. Stroke patients who could finish one or more tasks were categorized as having PPH, while those could not finish any task were classified as having CPH.

### 2.2 Behavioral assessment

The Hand and Wrist subscale of the Fugl‐Meyer Assessment (FMA‐HW) was used to evaluate paretic hand performance in all stroke patients before fMRI scanning.^8^ The FMA‐HW subscale, which was regarded as the primary measurement, consists of a wrist section (five items) and a hand section (seven items), with a possible score ranging from 0 to 24.

### 2.3 Collection of imaging data

Imaging data were acquired using a 3‐Tesla scanner (SIEMENS Trio, Germany). T1‐weighted images were collected using an MPRAGE sequence: 192 sagittal slices, 1 mm slice thickness, 0.5 mm gap, 1900/3.42/900 ms repetition time/echo time/inversion time, 240 × 240 field of view, 9° flip angle, and 256 × 256 matrix size. T2‐weighted images were collected using a TSE sequence: 30 axial slices, 5 mm slice thickness, without gap, 6000/93 ms repetition time/echo time, 220 × 220 field of view, 120° flip angle, and 320 × 320 matrix size. Resting‐state functional imaging data were acquired using an EPI sequence: 30 axial slices, 4 mm slice thickness, 0.8 mm gap, 2000/30 ms repetition time/echo time, 220 × 220 field of view, 90° flip angle, 64 × 64 matrix size, 240 volumes, and scanning time 8:06 (m:ss). During scanning, each participant was asked to keep their eyes closed and mind relaxed and to not move to the greatest extent.

### 2.4 Mapping lesion overlap

**FIGURE 1:**
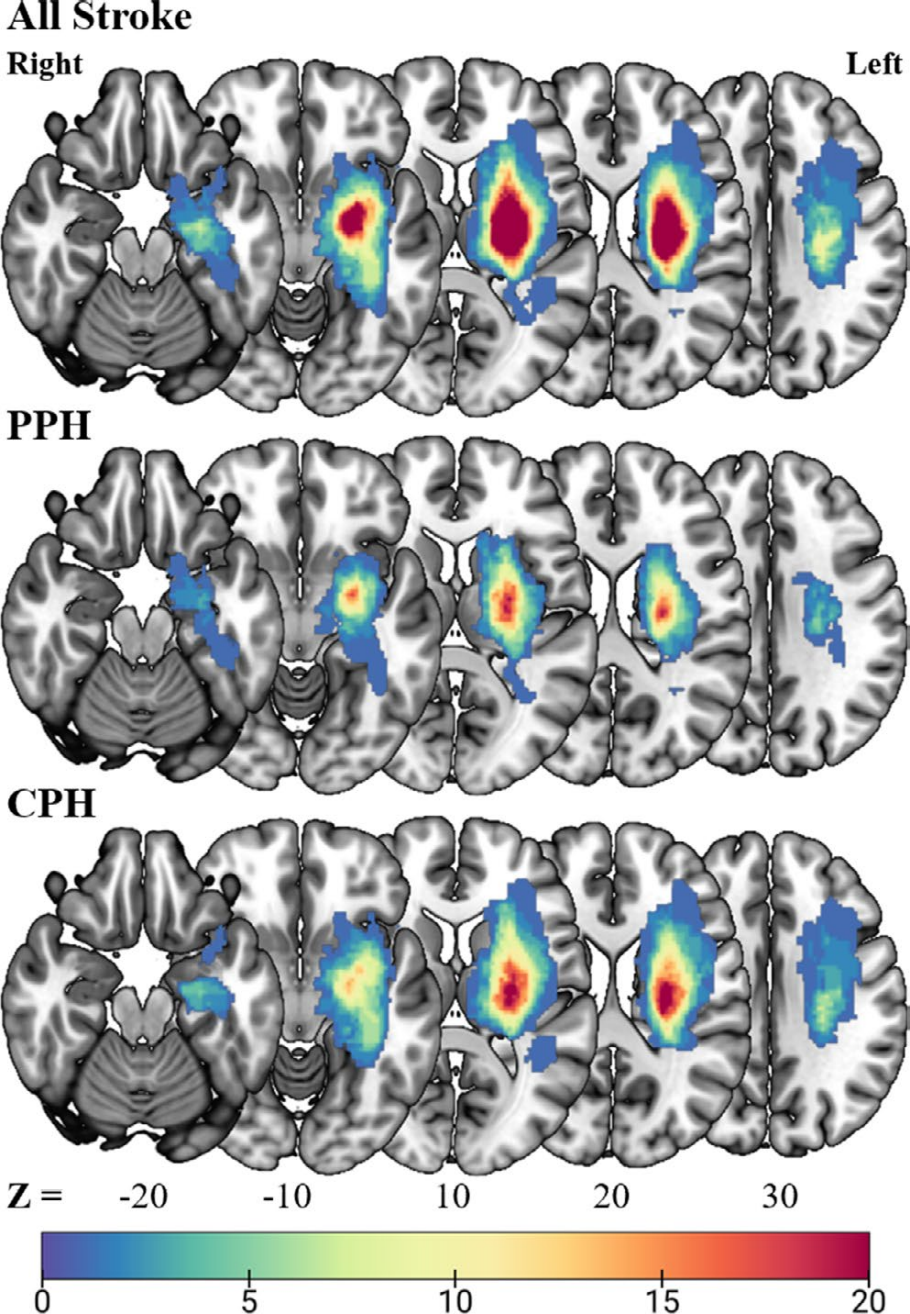
Lesion overlap map for all stroke, PPH, and CPH patients. The color bar indicates the frequency of patients having lesions in each voxel in the left (ipsilesional) hemisphere. CPH, completely paretic hand; PPH, partially paretic hand

We first used MRIcron software (https://people.cas.sc.edu/rorden/mricron/install.html) to delineate the lesion profiles of each stroke patient on T2‐weighted images (see Supporting Materials). Then, the T2‐weighted lesion masks of all stroke patients were standardized to the MNI space. Finally, we summed each resampled lesion mask with a resolution of 1 × 1 × 1 mm^3^ to establish the lesion map (Figure 1).

### 2.5 Imaging data preprocessing

We employed DPABI software (http://rfmri.org/DPABI) to preprocess the resting‐state functional imaging data.^34^ The processing steps involved (a) deletion of the first ten volumes, (b) correction of slice timing, (c) realignment of head motion, (d) standardization to the MNI space using the unified segmentation of structural images, (e) spatial smoothing (FWHM = 6 mm), (f) linear detrending, and (g) bandpass filtering (0.01–0.1 Hz). Finally, we regressed out the six head motion parameters, global mean signal, cerebrospinal fluid signal, and white matter signal. During image preprocessing, no participants were discarded based on the predefined criteria of head motion (exceeding 2 mm/degree). To eliminate the influences of head motion on the EC results,^35^ we regressed out the framewise displacement in all subsequent between‐group statistical analyses.

### 2.6 Definition of the SMC subregions

**FIGURE 2:**
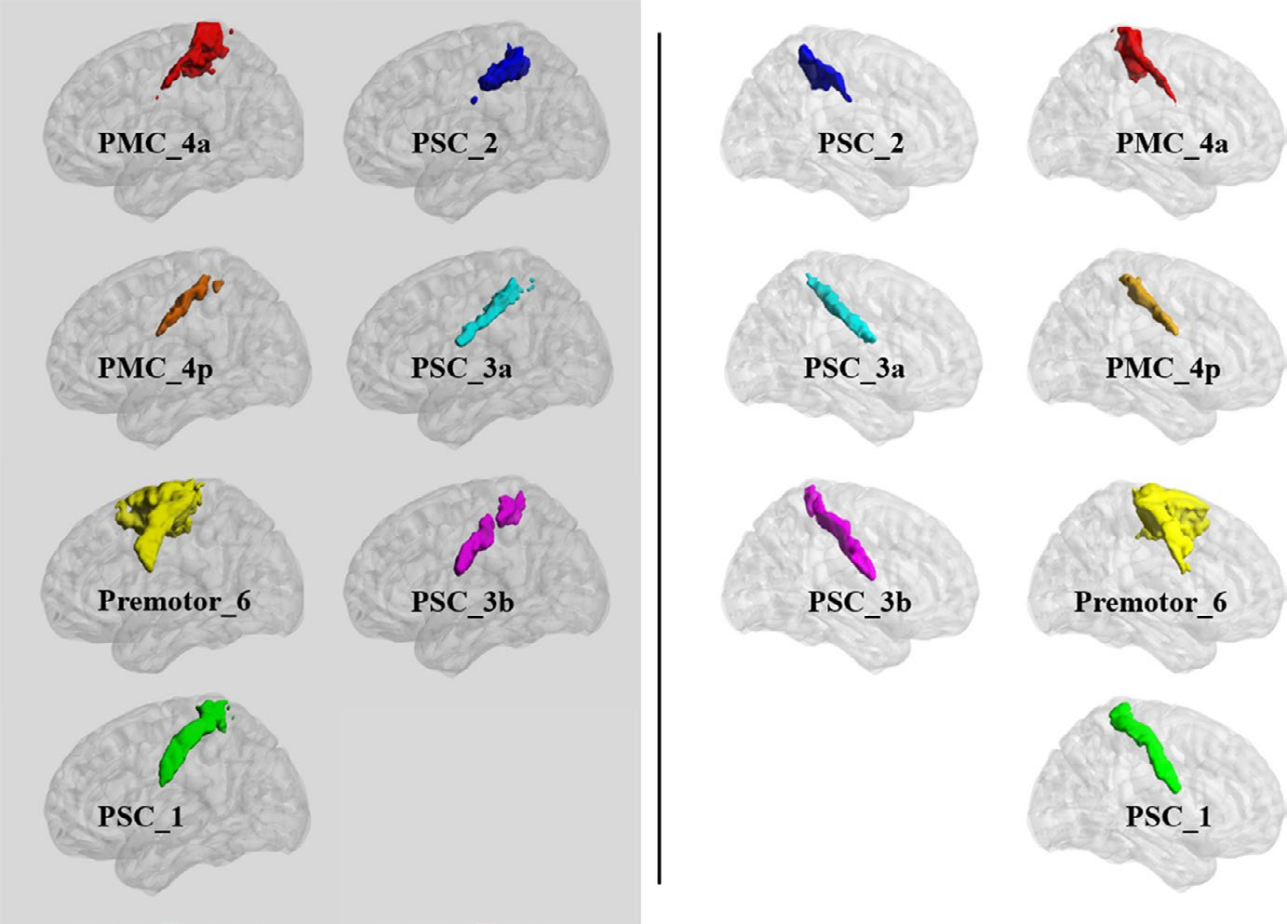
In total, fourteen seeds were defined by sensorimotor subregions based on the cytoarchitectonic atlas. The left panel shows seven ipsilesional (left) hemisphere seeds, and the right panel shows seven contralesional (right) hemisphere seeds

We defined seven SMC subregions in the ipsilesional and contralesional hemispheres on the basis of the probabilistic cytoarchitectonic atlas, as integrated in SPM12 software (https://www.fil.ion.ucl.ac.uk/spm/software/spm12/).^33^ For each hemisphere, the seven SMC subregions included premotor_6, PMC_4a, PMC_4p, primary somatosensory cortex_1 (PSC_1), PSC_2, PSC_3a, and PSC_3b (Figure 2).

### 2.7 EC analysis of the SMC subregions

The Granger causality analysis module within REST software (http://restfmri.net/forum/REST_V1.8) was used to generate voxelwise seed‐based EC maps.^36^ We employed the vector autoregression model to perform the Granger causality analysis. First, we performed a bivariate coefficient Granger causality analysis to obtain the EC maps for all participants and then converted these maps to z‐values using Fisher's conversion. Next, we employed one‐sample *t*‐tests to obtain the group EC patterns of each of the seven SMC subregions in each hemisphere.

### 2.8 Statistical analysis

To analyze the baseline data of all participants, SPSS software (version 25.0, IBM Inc.) was used. We first performed the Shapiro–Wilk test to assess the normality of all continuous variables (age, duration of illness, lesion volumes, FMA‐HW score, and framewise displacement). Then, we found that only age was distributed normally and thus was analyzed by the two independent samples *t*‐test, while the other variables were distributed abnormally and were analyzed by the Mann–Whitney test. For the sex ratio and stroke type, we employed the chi‐square test to analyze the differences between groups. To infer the EC differences between groups, we used two‐sample *t*‐tests to explore disrupted EC between all stroke patients and healthy participants as well as between CPH patients and PPH patients with sex, age and framewise displacement as covariates. The AlphaSim method with a *p* < 0.0001 was adopted to perform multiple comparisons (voxel *p* = 0.001, cluster ≥ 43, FWHM = 7.9 mm, with a gray matter mask). Finally, we used multiple linear regression analysis within SPM12 software (https://www.fil.ion.ucl.ac.uk/spm/software/spm12/) to explore associations between the EC patterns of each subregion and the FMA‐HW scores in all stroke patients with sex, age, and framewise displacement as covariates. We also performed the same analysis in both the PPH and CPH subgroups. The AlphaSim method with a *p* < 0.01 was adopted to perform multiple comparisons (voxel *p* = 0.001, cluster ≥ 19, FWHM = 7.0 mm, with a gray matter mask). For surviving brain regions from the multiple linear regression analysis, we first extracted the EC values within these regions and then correlated the EC values of each surviving region with the FMA‐HW scores in all stroke patients, PPH patients, and CPH patients by using Pearson correlation analysis. For the visualization of all results, we used the 3D surface of BrainNet Viewer software (www.nitrc.org/projects/bnv).^37^

## 3 RESULTS

### 3.1 Basic demographic and clinical data

Sixty‐five patients with left subcortical chronic stroke (32 PPH vs. 33 CPH) and forty healthy controls were recruited. We found that there were significant differences in the sex ratio (*p* = 0.009) and no significant differences in age (*p* = 0.671) or framewise displacement (*p* = 0.089) between stroke patients and healthy controls. Furthermore, we found that there were no significant differences in age (*p* = 0.811), sex ratio (*p* = 0.110), stroke type (*p* = 0.540), duration of illness (*p* = 0.305) or framewise displacement (*p* = 0.823) between the PPH and CPH subgroups. However, the lesion volumes of the CPH patients were significantly larger than those of the PPH patients (*p* = 0.006), and the FMA‐HW scores of the PPH patients were significantly higher than those of the CPH patients (*p* < 0.001) (Table 1).

**TABLE 1:** Demographic and clinical data of participants recruited in this study

Baseline characteristics	Study 1		*p* value	Study2		*p* value
	All stroke	Controls		PPH	CPH	
Age (years)^b^	55.89 ± 9.71	55.12 ± 7.57	0.671	56.19 ± 10.53	55.60 ± 9.00	0.811
Sex (male:female)^a^	54:11	24:16	**0.009**	29:3	25:8	0.110
Hand dominance	Right	Right	—	Right	Right	—
Stroke type (ischemic:hemorrhagic)^a^	30:35	—	—	16:16	14:19	0.540
Duration of illness (months)	14.70 ± 16.07	—	—	15.31 ± 14.87	14.12 ± 17.36	0.305
Lesion hemisphere (left:right)	Left	—	—	Left	Left	—
Lesion location	Subcortical	—	—	Subcortical	Subcortical	—
Lesion volume (ml)	12.78 ± 9.50	—	—	9.45 ± 5.57	16.00 ± 11.33	**0.006**
FMA‐HW score	6.17 ± 6.67	—	—	11.25 ± 6.15	1.24 ± 1.22	**<10^−9^**
Framewise displacement (mm)	0.14 ± 0.08	0.11 ± 0.05	0.089	0.13 ± 0.07	0.14 ± 0.10	0.823

Note

Values expressed as the mean ± SD; the superscript a indicates the chi‐square test, b indicates two independent sample *t*‐test, and all others are Mann–Whitney tests.

Abbreviations: CPH, completely paretic hand; FMA‐HW, Fugl‐Meyer Assessment of Hand and Wrist; PPH, partially paretic hand.

The bold value used to highlight the significant p values.

### 3.2 Differences in EC between stroke patients and controls

**FIGURE 3:**
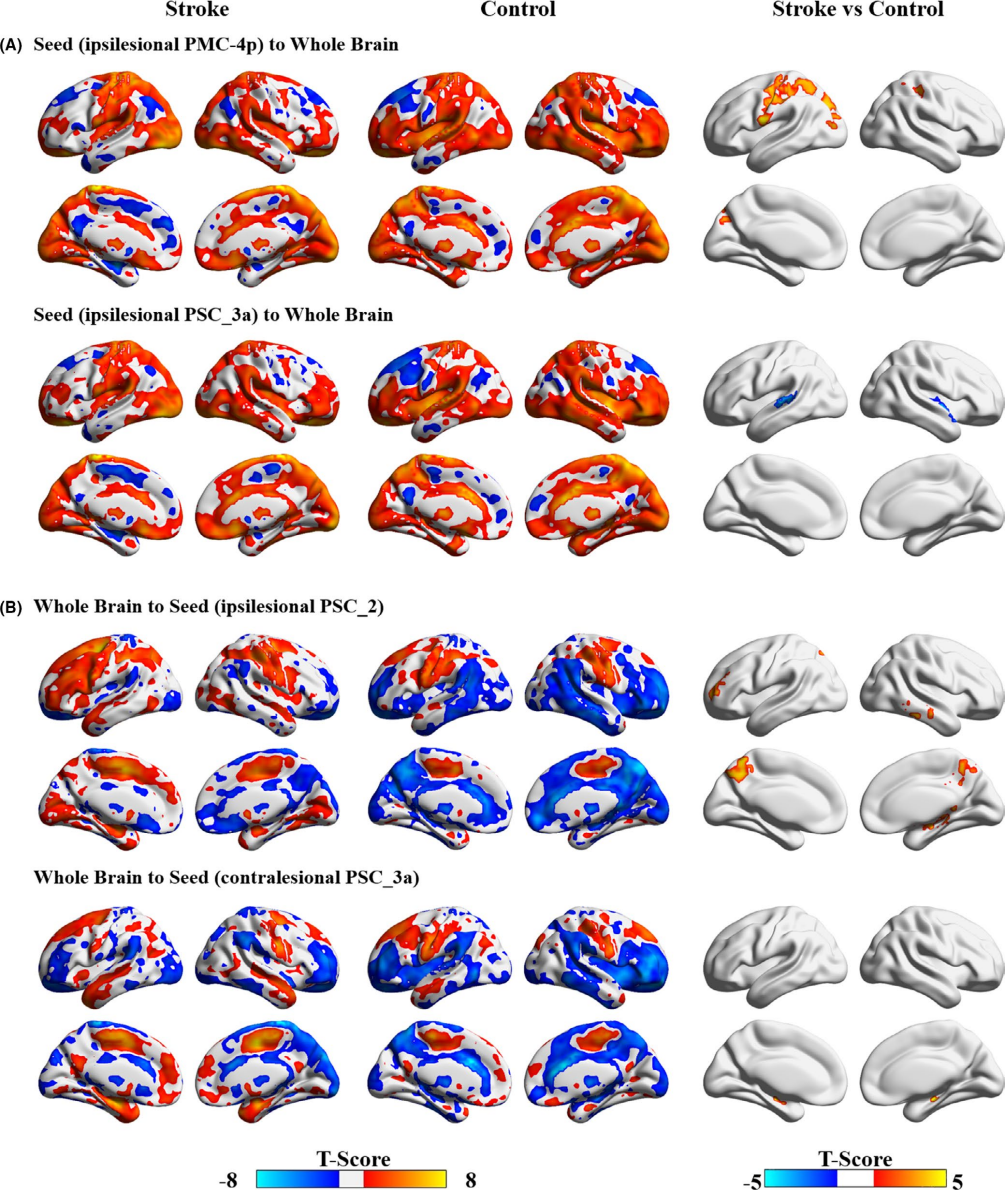
Disrupted effective connectivity in stroke patients compared with healthy controls. (A) Group differences in effective connectivity from seeds to the whole brain. (B) Group differences in effective connectivity from the whole brain to seeds. AlphaSim corrected: *p* < 0.0001. PMC, primary motor cortex; PSC, primary somatosensory cortex

Compared with controls, stroke patients demonstrated increased EC from the ipsilesional PMC_4p to the ipsilesional precentral gyrus, contralesional postcentral gyrus, and ipsilesional middle occipital cortex, from the bilateral precuneus, contralesional middle temporal gyrus, contralesional hippocampus, and ipsilesional middle frontal gyrus to the ipsilesional PSC_2, and from the bilateral hippocampus and ipsilesional precuneus to the contralesional PSC_3a. Additionally, compared with controls, stroke patients demonstrated decreased EC from the ipsilesional PSC_3a to the bilateral superior temporal gyrus (Table 2, Figure 3).

**TABLE 2:** Comparison of EC between stroke patients and healthy controls and between stroke subgroups

Disrupted effective connectivity	MNI			Cluster	*t* value
	*X*	*Y*	*Z*		
Stroke > Control					
Ipsilesional PMC_4p to ipsilesional precentral gyrus	−36	−18	36	730	5.78
Ipsilesional PMC_4p to contralesional postcentral gyrus	42	−36	54	61	3.96
Ipsilesional PMC_4p to ipsilesional middle occipital cortex	−24	−78	36	222	5.00
Ipsilesional precuneus to ipsilesional PSC_2	−3	−54	48	219	5.37
Contralesional precuneus to ipsilesional PSC_2	12	−54	48	127	4.38
Ipsilesional middle frontal gyrus to ipsilesional PSC_2	−36	36	18	137	4.52
Contralesional middle temporal gyrus to ipsilesional PSC_2	54	−30	−15	77	5.26
Contralesional hippocampus to ipsilesional PSC_2	21	−33	−9	145	5.09
Ipsilesional hippocampus to contralesional PSC_3a	−36	0	−15	83	6.82
Contralesional hippocampus to contralesional PSC_3a	30	−6	−15	116	4.68
Ipsilesional precuneus to contralesional PSC_3a	−9	−51	60	103	5.42
Stroke < Control					
Ipsilesional PSC_3a to ipsilesional superior temporal gyrus	−63	−24	3	65	−5.80
Ipsilesional PSC_3a to contralesional superior temporal gyrus	42	−3	−12	144	−4.81
PPH < CPH					
Ipsilesional inferior parietal lobe to ipsilesional premotor_6	−36	−60	42	34	−4.37
Ipsilesional inferior parietal lobe to ipsilesional PSC_1	−36	−57	39	55	−4.32
Ipsilesional inferior temporal gyrus to ipsilesional PSC_1	−57	−30	−24	47	−5.22
Contralesional middle frontal cortex to ipsilesional PSC_1	42	42	15	30	−4.17

Abbreviations: CPH, completely paretic hand; PMC, primary motor cortex; PPH, partially paretic hand; PSC, primary somatosensory cortex.

### 3.3 Brain‐behavior correlations including all stroke patients

**FIGURE 4:**
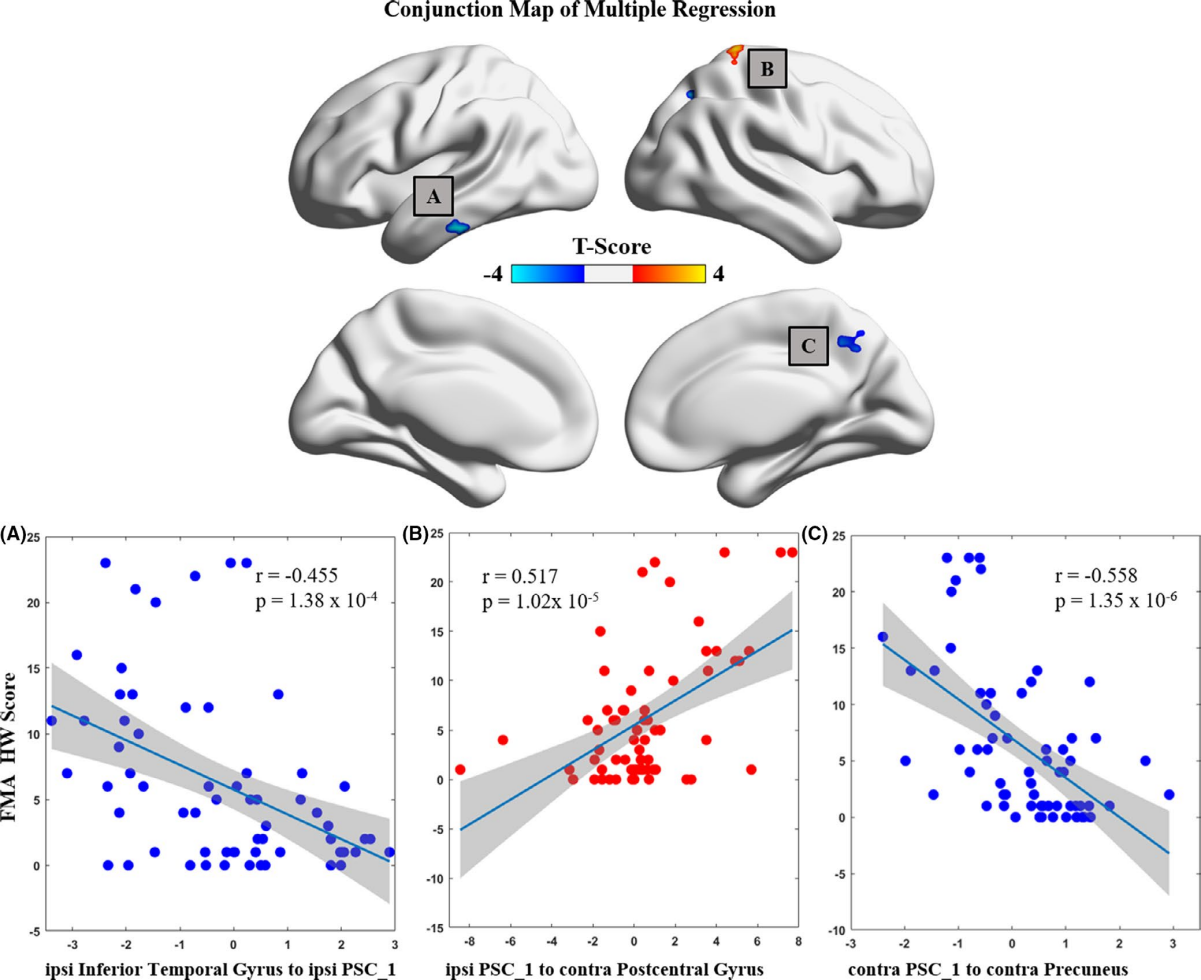
Correlations between connectivity patterns and paretic hand performance for all stroke patients. AlphaSim corrected: *p* < 0.01. Contra, contralesional; FMA‐HW, Fugl‐Meyer Assessment of Hand and Wrist; ipsi, ipsilesional; PSC, primary somatosensory cortex

Specifically, the FMA‐HW scores were negatively related to the EC values of the contralesional PSC_1 to the contralesional precuneus (*r* = −0.558, *p* < 0.001) and the ipsilesional inferior temporal gyrus to the ipsilesional PSC_1 (*r* = −0.455, *p* < 0.001). However, the FMA‐HW scores were positively related to the EC values of the ipsilesional PSC_1 to the contralesional postcentral gyrus (*r* = 0.517, *p* < 0.001) (Figure 4).

### 3.4 Distinct EC patterns between stroke patients with CPH and PPH

**FIGURE 5:**
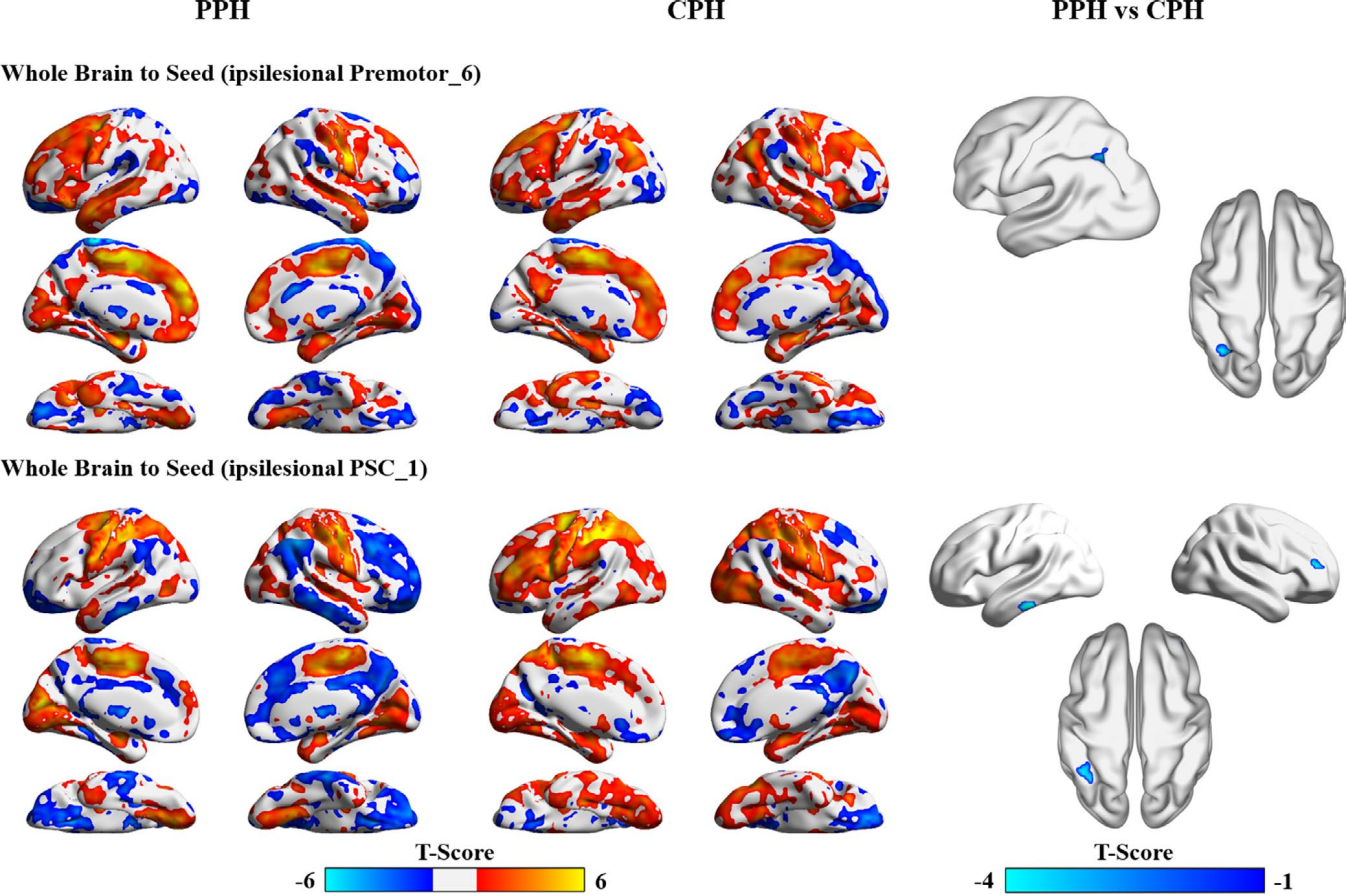
Disrupted effective connectivity between stroke patients with partially and completely paretic hands. AlphaSim corrected: *p* < 0.0001. CPH, completely paretic hand; PPH, partially paretic hand; PSC, primary somatosensory cortex

Compared with the CPH patients, the stroke patients with PPH demonstrated decreased EC from the ipsilesional inferior parietal lobe to the ipsilesional premotor_6 and ipsilesional PSC_1 and from the ipsilesional inferior temporal gyrus and contralesional middle frontal cortex to the ipsilesional PSC_1 (Table 2, Figure 5).

### 3.5 Brain‐behavior correlations across stroke subgroups

**FIGURE 6:**
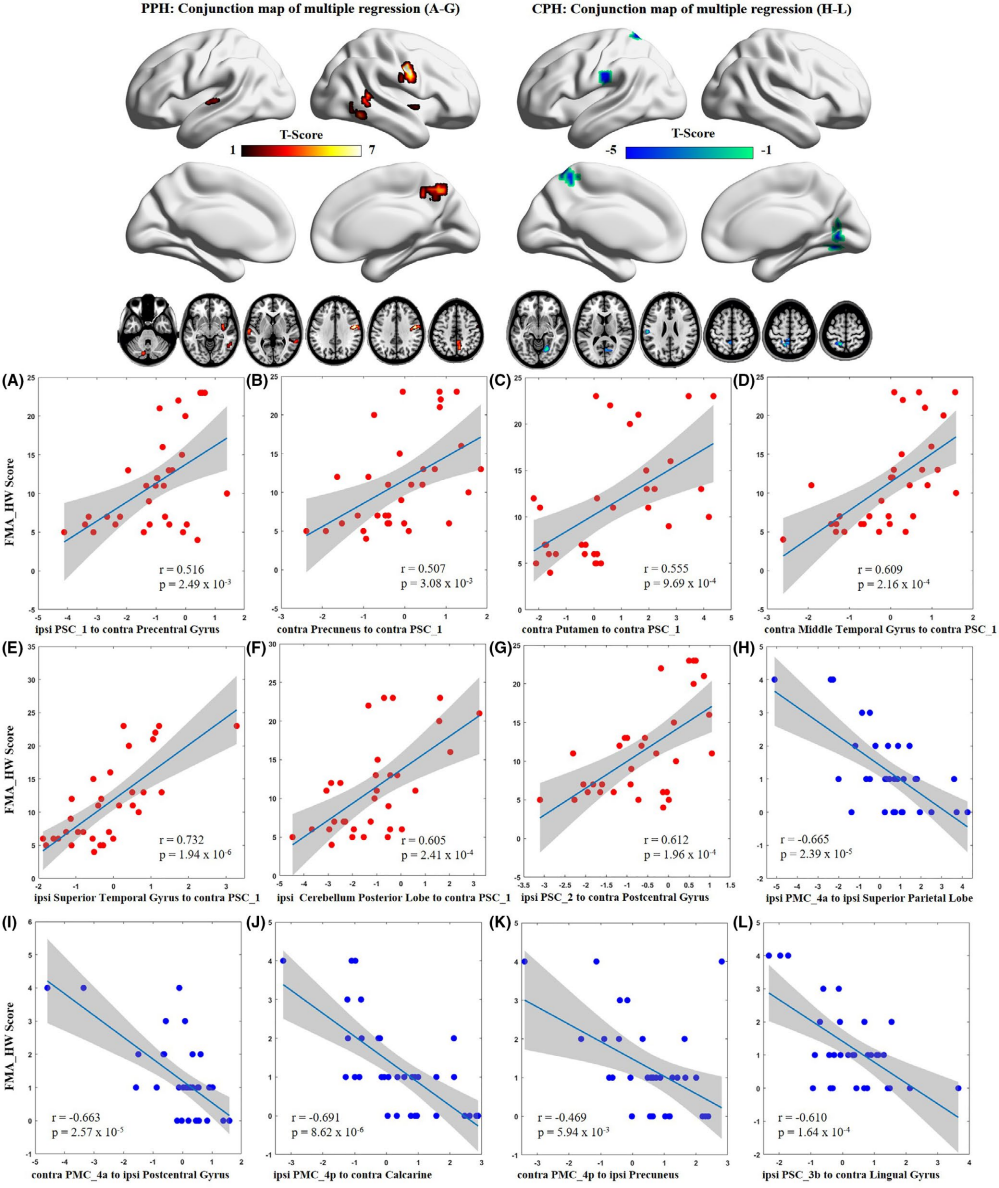
Dissociated correlations between connectivity patterns and different hand outcomes in stroke subgroups. AlphaSim corrected: *p* < 0.01. Contra, contralesional; CPH, completely paretic hand; FMA‐HW, Fugl‐Meyer Assessment of Hand and Wrist; ipsi, ipsilesional; PMC, primary motor cortex; PPH, partially paretic hand; PSC, primary somatosensory cortex

The stroke patients with PPH showed only positive brain‐behavior correlations. Specifically, the FMA‐HW scores were positively related to the EC values of the ipsilesional PSC_1 to the contralesional precentral gyrus (*r* = 0.516, *p* = 0.002), the contralesional precuneus (*r* = 0.507, *p* = 0.003), contralesional putamen (*r* = 0.555, *p* < 0.001), contralesional middle temporal gyrus (*r* = 0.609, *p* < 0.001), ipsilesional superior temporal gyrus (*r* = 0.732, *p* < 0.001), and ipsilesional cerebellum posterior lobe (*r* = 0.605, *p* < 0.001) to the contralesional PSC_1, and the ipsilesional PSC_2 to the contralesional postcentral gyrus (*r* = 0.612, *p* < 0.001). In contrast, the stroke patients with CPH showed only negative brain‐behavior correlations. Specifically, the FMA‐HW scores were negatively related to the EC values of the ipsilesional PMC_4a to the ipsilesional superior parietal lobe (*r* = −0.665, *p* < 0.001), the contralesional PMC_4a to the ipsilesional postcentral gyrus (*r* = −0.663, *p* < 0.001), the ipsilesional PMC_4p to the contralesional calcarine (*r* = −0.691, *p* < 0.001), the contralesional PMC_4p to the ipsilesional precuneus (*r* = −0.469, *p* = 0.005), and the ipsilesional PSC_3b to the contralesional lingual gyrus (*r* = −0.610, *p* < 0.001) (Figure 6).

## 4 DISCUSSION

The challenge in post‐stroke neuroimaging studies is to identify the intervention targets^38^ and predict the long‐term outcomes.^39^ Using Granger causality analysis of resting‐state fMRI data in chronic stroke patients, we found that the EC patterns of SMC subregions were selectively disrupted and correlated with hand dysfunction. Most importantly, the correlations between EC patterns and hand performance in stroke patients with PPH were positive, while those in stroke patients with CPH were negative. These findings indicate that injury of the subcortical motor pathway results in specific disruption of sensorimotor circuits, which may inspire the development of neuroimaging biomarkers^40^ and stimulation targets^41^ in neurorehabilitation practice after chronic stroke.

### 4.1 Disrupted sensorimotor circuits following chronic stroke

Usually, stroke patients with severe motor impairments exhibit hyperactivation of the sensorimotor system.^42^, ^43^, ^44^ In fact, activation in the ipsilesional premotor and primary motor areas^45^ without recruitment of contralesional activity^46^ is related to good motor outcomes. However, chronic stroke patients who receive bilateral arm training demonstrate a recovery‐associated increase in activation in the contralesional SMC.^47^ Recent connectivity studies have suggested that recovery of motor function is driven by decreased interhemispheric influences from the contralesional PMC to the ipsilesional PMC following stroke.^13^, ^48^ Additionally, a previous electrophysiological study also revealed that overactivation of the contralesional PMC might inhibit the motor output of the ipsilesional PMC in chronic stroke patients.^49^ Contrary to these findings,^13^, ^48^, ^49^ we demonstrated here that stroke patients show increased EC from the ipsilesional PMC_4p to the ipsilesional precentral gyrus and contralesional postcentral gyrus, and paretic hand performance is positively related to the EC strength of the ipsilesional PSC_1 to the contralesional postcentral gyrus. Our results verify but also extend previous findings, highlighting the importance of interhemispheric disturbances among sensorimotor regions for hand dysfunction observed in chronic stroke patients.^9^, ^22^ Furthermore, these data suggest a functional relevance of the disrupted influences from the ipsilesional to contralesional somatosensory cortices due to the strong brain‐behavior correlations.

Stroke patients with motor deficits typically show recruitment of non‐motor regions (eg, the prefrontal, parietal, and temporal lobes), with the consensus that greater activation of non‐motor areas leads to poorer functional recovery.^50^ The anterior precuneus is closely related to sensorimotor processing.^51^ One longitudinal study indicated that hyperactivation in the precuneus is correlated with slower motor recovery following stroke.^52^ Here, we demonstrated that not only task‐related activation of the precuneus but also EC strength from the bilateral precuneus to the ipsilesional PSC_2 is increased in chronic stroke patients. Furthermore, EC strength from the contralesional PSC_1 to the contralesional precuneus is negatively correlated with paretic hand performance. The precuneus has extensive connections with the SMC system, which plays important roles in visual goal‐directed hand movements.^53^ Thus, our data suggest that the exchange of information between the somatosensory cortex and precuneus is needed for chronic stroke patients to support visual processing during affected hand movements. Complex motor tasks, for example, novel and skilled sequential hand movements, often require audiomotor processing support from the bilateral temporal gyrus.^54^ A previous study found that increased spontaneous activity in the contralesional superior temporal gyrus is negatively correlated with motor dysfunction after chronic stroke.^10^ Interestingly, in this study, we demonstrated that stroke patients display decreased EC from the ipsilesional PSC_3a to the bilateral superior temporal gyrus, and paretic hand performance is negatively related to EC strength from the ipsilesional inferior temporal gyrus to the ipsilesional PSC_1. Considering the supporting role of the temporal gyrus in complex sensorimotor processing, our data suggest that disrupted interactions between temporal and sensory cortices might impair audiomotor coordination during affected hand movements following chronic stroke. The hippocampus is involved in motor learning consolidation, which could optimize subsequent behavior.^55^ After rehabilitation, chronic stroke patients show increased gray matter volume within the bilateral hippocampus^56^ and strengthened functional coupling between the ipsilesional inferior parietal lobe and bilateral parahippocampal gyrus,^57^ and these neuroplastic changes are positively correlated with motor improvements. Here, we demonstrated that EC strength from the bilateral hippocampus to the contralesional PSC_3a is increased in chronic stroke patients. Our data suggest that the hippocampus might learn and store the missing sensory input originating from the paretic hand to compensate for the information processing of the somatosensory cortex. Collectively, from the perspective of functional integration, our study expands the previous findings that disrupted causal interactions among the SMC, precuneus, temporal cortex, and hippocampus might underlie the pathophysiological mechanisms of hand dysfunction after chronic stroke.

### 4.2 Differently disrupted sensorimotor circuits between PPH and CPH patients

It is well known that the premotor cortex gives rise to the cortico‐reticulospinal tract and is specifically related to proximal movement,^58^ which is a good substitution for hand function recovery after mild to moderate stroke.^50^ The premotor cortex involves transferring sensory stimuli into motor programs,^59^ and their structural pathways are connected to the parietal lobe associated with motor output.^60^ In chronic stroke patients, activation of the contralesional dorsal premotor cortex is increased, and interference of their activity with transcranial magnetic stimulation can deteriorate motor performance.^61^, ^62^ Furthermore, strengthened EC between the anterior intraparietal sulcus and PMC within the ipsilesional hemisphere is evident in the well‐recovered subgroup.^63^ These findings emphasize the important roles of the premotor cortex and its interactions with the parietal lobe in motor dysfunction after chronic stroke. We extended earlier findings by revealing that stroke patients with CPH show increased EC from the ipsilesional inferior parietal lobe to the ipsilesional premotor_6 and PSC_1. Therefore, we speculate that the facilitatory influences from the frontoparietal areas to the SMC system in the lesioned hemisphere might represent greater top‐down control over the SMC to assist motor execution after chronic stroke.^32^ The prefrontal cortex and temporal gyrus play crucial roles in higher‐order planning as well as audiomotor coordination during complex hand movement.^20^ For stroke patients with acute striatal infarcts, increased gray matter thickness has been found in the frontal and temporal cortices but not the motor cortex.^64^ Another longitudinal task fMRI study also demonstrated that reduced activation in the prefrontal and temporal cortices over time is related to motor recovery following stroke.^65^ Furthermore, a recent functional connectivity study found that increased coupling between the ipsilesional PMC and the contralesional middle frontal gyrus could predict long‐term motor outcomes after stroke.^25^ Well in line with these findings, we found that stroke patients with CPH demonstrate increased EC from the contralesional middle frontal cortex and ipsilesional inferior temporal gyrus to the ipsilesional PSC_1. Recent evidence has revealed that persistent recruitment of remote cortices during spontaneous recovery is associated with poor motor outcomes.^66^ Thus, the convergently increased EC from these non‐motor regions to the ipsilesional SMC subregions might contribute to maintaining the final hand outcomes, while it indicates an unoptimizable process of recovery‐associated neuroplasticity in severely stroke patients.

### 4.3 Dissociated sensorimotor circuits correlated with different hand outcomes

Two different functional reorganizations have been reported after rehabilitation in chronic stroke patients.^67^ Specifically, patients with intact or damaged PMCs and their descending motor pathway show decreased or increased activation in the ipsilesional SMC. Interestingly, we also found two brain‐behavior correlations in which paretic hand performance is positively correlated with EC patterns in stroke patients with PPH but negatively correlated with EC patterns in CPH patients. In stroke patients with PPH, convergence of positive correlations to the contralesional PSC_1 indicates that this region holds a highly important function within the sensorimotor network configurations (eg, brain hub) to drive motor recovery.^68^ However, in stroke patients with CPH, distributed negative correlations from non‐motor regions (eg, the visual cortex) may represent a compensatory cognitive strategy, for example, visuospatial processing, to sustain poor hand outcomes. Except for physiological processes (eg, decreased GABAergic inhibition and increased NMDA facilitation),^66^ excessive activation within the SMC system during paretic hand movement is primarily determined by the structural integrity of the corticospinal tract in stroke patients.^42^, ^43^ Therefore, we speculate that the different injury loads of the corticospinal tract between CPH and PPH patients may be the important reason for these dissociated positive and negative correlations.^69^

### 4.4 Limitations and future considerations

First, stroke patients show a heavy male predominance because endogenous estrogen can exert neuroprotective effects for premenopausal women away from a higher risk for stroke.^70^ To address this bias, we regressed out sex in the statistical analyses. Second, this was not a longitudinal/interventional study, making it difficult to infer the dynamic evolution of EC patterns in SMC subregions during the process of motor recovery. Third, considering the enormous values of injured corticospinal tracts for predicting motor recovery in stroke patients,^7^, ^71^ it will be promising to combine structural and functional biomarkers for the prediction of treatment responses. Finally, although modulating bilateral PMC targets has been shown to be beneficial for stroke patients with hand dysfunction,^11^, ^13^, ^14^, ^15^, ^72^ future studies might consider additional targets found in this study (eg, the postcentral gyrus) to design neuromodulation experiments.^41^, ^73^

## 5 CONCLUSION

In this study, we systematically investigated EC patterns between sensorimotor subregions and the whole brain after chronic stroke. We found that large‐scale sensorimotor circuits are selectively disrupted and that dissociated motor‐related neurocircuitry is associated with different hand outcomes in chronic stroke patients, which has rarely been reported in previous studies. Our findings indicate that these disrupted sensorimotor circuits might be considered potential neuroimaging biomarkers and stimulation targets to repair lesion‐induced abnormal motor networks,^74^ which in turn, facilitate hand rehabilitation after chronic stroke.

## CONFLICTS OF INTEREST

The authors declare no competing interests.

## AUTHOR CONTRIBUTIONS

C.T. and Z.Z. developed and designed the study concept; C.T. coordinated and collected the data; F.L. conducted the data analyses under supervision from C.T. and Z.Z.; F.L. and C.T. wrote the initial draft; and all authors revised and confirmed the final version.

## Supporting information

Supplementary Material

Supplementary Material

## Data Availability

Data are available upon request from the corresponding authors.
