# Supplementary material for: Selectively disrupted sensorimotor circuits in chronic stroke with hand dysfunction
Source: CNS Neurosci Ther. 2022 Jan 10;28(5):677–89. doi: 10.1111/cns.13799 (PMC8981435; doi:10.1111/cns.13799)
Supplement: Supplementary file 1 — Supplementary Material [file CNS-28-677-s001.docx]

**Fig. A. 1** The lesion location of each PPH and CPH patient is shown in T2-weighted axial images. Left indicates the ipsilesional hemisphere.

**
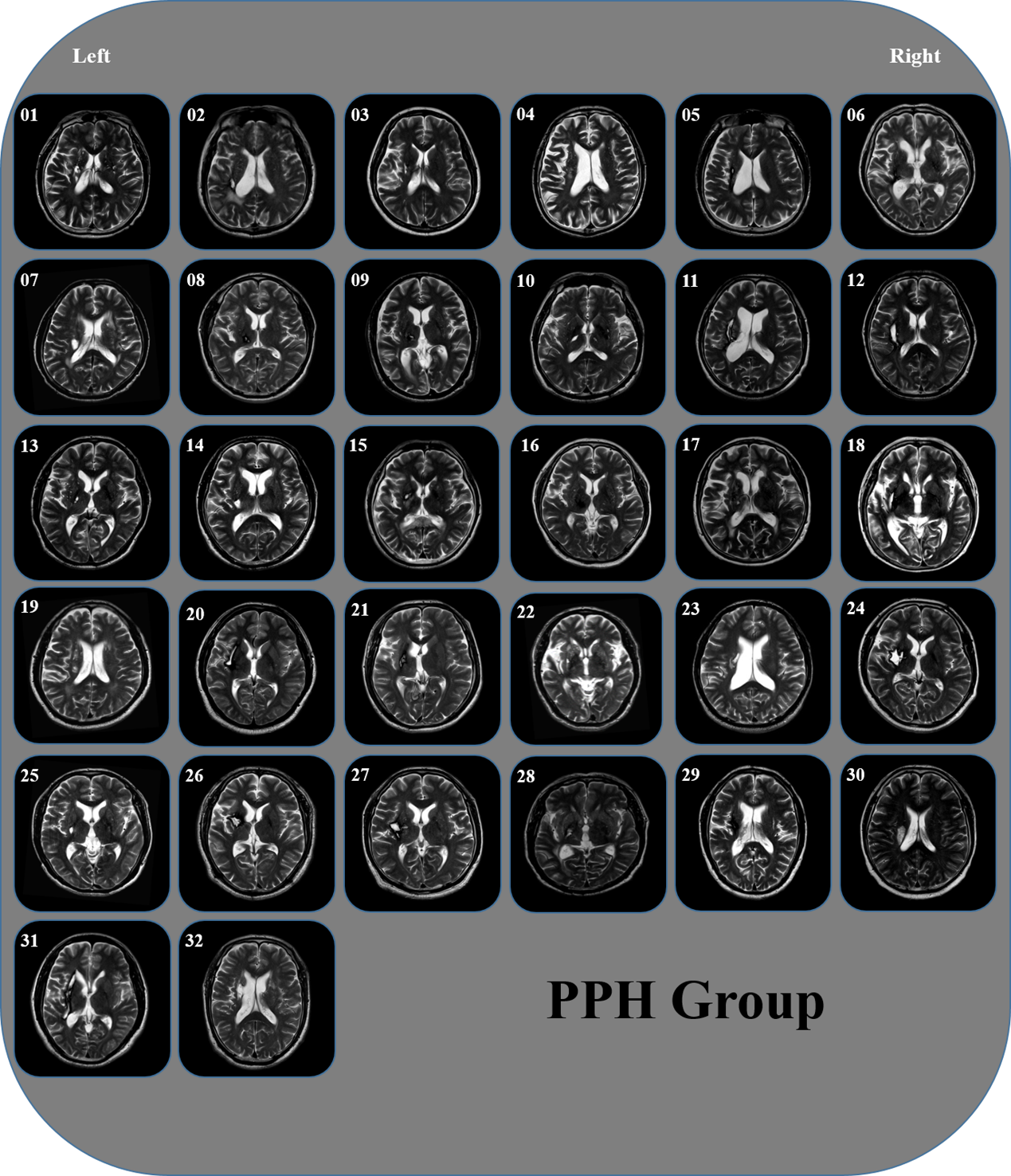
**

**
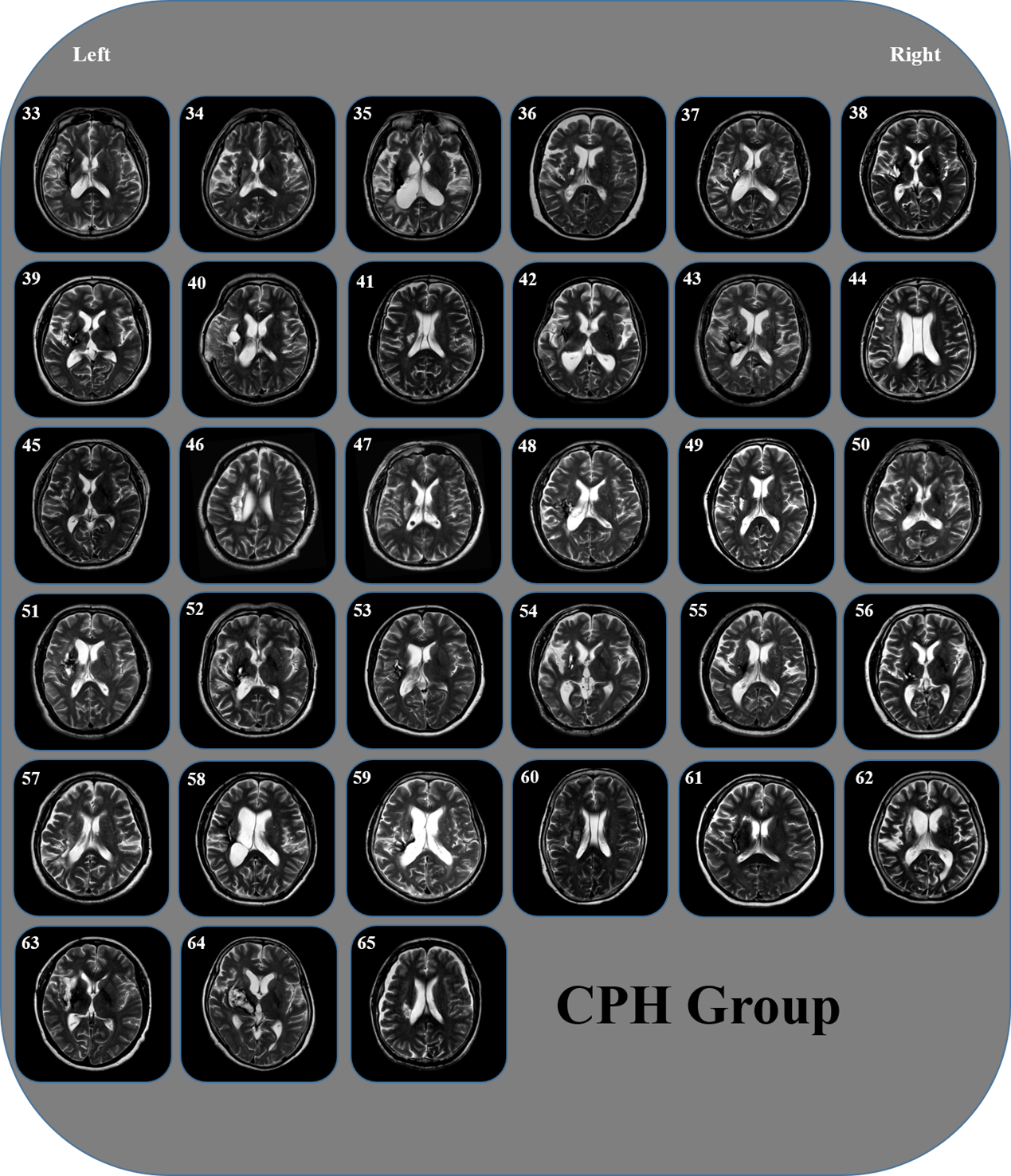
**
