# Supplementary material for: Selectively disrupted sensorimotor circuits in chronic stroke with hand dysfunction
Source: CNS Neurosci Ther. 2022 Jan 10;28(5):677–89. doi: 10.1111/cns.13799 (PMC8981435; doi:10.1111/cns.13799)
Supplement: Supplementary file 2 — Supplementary Material [file CNS-28-677-s002.docx]

**Table A.1** Paralyzed Hand Function Assessment Scale.

| **Action items** | **Illustration** | **Hand function classification** | **Evaluation criteria** |
| --- | --- | --- | --- |
| 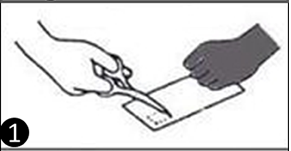 | The affected hand stabilizes a piece of paper on the table, and the unaffected hand uses a shear to cut the paper. | Disabled hand | Could not complete any activities. |
| 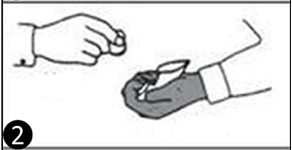 | The affected hand holds a wallet, and the unaffected hand takes a coin from the wallet. | Assistant hand C | Finished one of the five activities. |
| 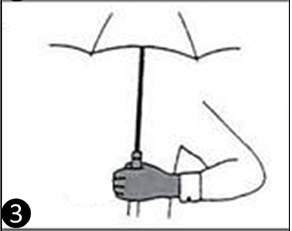 | The affected hand holds an unfolded umbrella in the air for at least 10 seconds. | Assistant hand B | Finished two of the five activities. |
|  |  | Assistant hand A | Finished three of the five activities. |
| 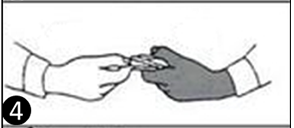 | The affected hand controls a nail scissor to trim nails of the unaffected hand. | Practical hand B | Finished four of the five activities. |
| 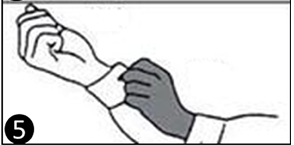 | The affected hand buttons the cuff of the unaffected side. | Practical hand A | Completed all of the five activities. |

**Note.** The hand in dark denotes the affected hand.
